# Utilizing PDSA Cycle in Implementing a Chest Pain Accelerated Diagnostic Protocol

**DOI:** 10.51894/001c.6436

**Published:** 2017-12-19

**Authors:** Gretchen Breckner, Jennifer Walker, Karen Hanley, Nikolai Butki

**Affiliations:** 1 McLaren Oakland, Pontiac, MI

**Keywords:** heart score, qi/ps, patient safety, quality improvement

## Abstract

**CONTEXT:**

The authors in the Emergency Department (ED) at McLaren Oakland utilized the Plan-Do-Study-Act (PDSA) model to implement, evaluate and incrementally modify a Chest Pain Accelerated Diagnostic Protocol (CPADP) using the History, EKG, Age, Risk Factors, Troponin (HEART) Score at their institution. The objective of this study was to evaluate the ability of patients who presented to the ED with chest pain and fell into the low risk category based on their HEART Score to receive adequate outpatient follow-up for their chest pain.

**METHODS:**

Modifying protocols implemented at other institutions, in 2016 the authors developed CP-ADP utilizing the HEART Score to risk-stratify patients presenting to the ED with chest pain as low, moderate or high risk for major adverse cardiac events (MACE). Patients identified as low risk were offered the options of hospital observation or being discharged home with outpatient follow-up within seven days. Patients who were risk-stratified into the medium or high risk for MACE were admitted into the in-patient setting for cardiac evaluation. Once implemented, the protocol was evaluated to measure patient follow-up within thirty days.

**RESULTS:**

During a five-month period, 50 patients presenting to the ED with chest pain were risk-stratified as low risk for adverse cardiac events and opted for discharge from the ED to follow-up in the outpatient setting. A total of 18 patients were lost to follow up, and two patients subsequently returned to the ED for further evaluation of their chest pain and were admitted to the inpatient setting. These two patients were not included in the analysis. Thirty patients were successfully contacted by telephone 30 days postdischarge. Of those 30 patients contacted, none experienced any MACE events. However, only 14 (47%) low risk patients followed up with a primary care provider or cardiologist and only four (13%) received provocative cardiac testing (i.e., stress testing).

**CONCLUSIONS:**

Only 47% of patients discharged from the ED received outpatient follow-up and only 13% received cardiac testing. As a result of the study, the multi-disciplinary Chest Pain Committee has progressed to the Act ‘A’ step of the PDSA cycle to modify the authors’ protocol to ensure more clinically appropriate outpatient follow-up for patients discharged under the CP-ADP.

## INTRODUCTION

Myocardial infarction is one of the leading causes of death in the world.^1^ However, the majority of patients presenting to emergency departments (ED) with a chief complaint of chest pain are not found to have emergent cardiac causes for their chest pain.^2,3^ In fact, only about 10% of all patients presenting to emergency departments with chest pain are diagnosed with acute coronary syndrome (ACS), a condition earlier known as myocardial infarction or heart attack.^1^ As a result, the need for cardiac chest pain risk assessment tools and learning how to best use these tools, has become of increasing importance.

In addition, the high medical-legal risk that physicians feel associated with patients presenting to ED with chest pain highlights the need for a validated risk stratification tool to identify patients who can be safely discharged from the ED.^4^ The historical practice at McLaren Oakland hospital has been to admit patients presenting with chest pain to the hospital inpatient or observation units for further cardiac evaluation. This, however, is an expensive practice. It is estimated that admitting patients to the hospital only to result in testing with negative findings costs $5-10 billion annually in the United States.^5^

In addition to costs, there are also patient safety concerns associated with unnecessary hospital admissions. Patients admitted to hospitals for cardiac evaluation often receive an escalation in studies and testing which has led to overutilization of invasive procedures such as cardiac catheterization.^2^

In 2013, the HEART score was developed for use as a cardiac risk assessment tool.^1^ The HEART score produces a numeric score based on five patient factors: History, EKG findings, Age, Risk factors and Troponin results. The HEART score has been shown to accurately risk stratify patients for the potential to experience major adverse cardiac events (MACE).^1^ Particularly useful, the HEART score identifies patients at low risk who can be safely discharged from the ED, reducing unnecessary hospital admissions.^2,6^ The HEART score also identifies those patients at higher risk who require further workup and possible admission.^7^

In the HEART Score validation study, less than 1% of low risk patients experienced MACE within 30 days of their ED visits.^1^ Using this metric and a shared decision making between the patient and the physician regarding discharge options, low risk chest pain patients can be safely discharged from the ED with close primary care or cardiology outpatient follow up.

In 2016, the McLaren Oakland Chest Pain Committee was charged with designing an evidence-based protocol to discharge low risk chest pain patients that best fit the interest of the patients and institution. The interests of the patients include safe identification of serious cardiac diseases balanced with cost reduction, both direct in terms of payment and co-pays and indirect such as loss of work and productivity during the time in the hospital. The interests of the institution include providing thorough and safe patient care balanced with the costs of low reimbursed observation stays consuming resources.

The protocol was entitled Chest Pain Accelerated Diagnostic Protocol (CP-ADP). The Chest Pain Committee was also charged with the task of continuously evaluating and improving the protocol until it successfully met the best interests of patients and the institution. The Chest Pain Committee sought to develop a model that focused on Quality Improvement (QI), viewing health care as process and system improvement opportunities. The Chest Pain Committee chose the PDSA model to evaluate and modify the CP-ADP.^8,9^ (Appendix 1)

### Objectives

The goal of this Quality Improvement/Patient Safety (QI/PS) project was to utilize the PDSA model to design, evaluate and improve the McLaren Oakland CP-ADP to meet the needs of patients presenting to the ED with chest pain. The authors primarily wanted to ensure that chest pain patients who were opting for discharge with outpatient follow-up received appropriate outpatient follow-up and cardiac testing. Secondarily, the authors wanted to monitor the incidence of MACE in the patients discharged home under the CP-ADP protocol.

## METHODS

The McLaren Oakland Chest Pain Committee selected the HEART Score as the institutional cardiac risk stratification tool for patients presenting to the ED with a chief complaint of chest pain and who received a cardiac work up (i.e., basic lab work, troponins). The PDSA model was utilized to implement and modify the CP-ADP. (Appendix 2)

During the Plan stage of the cycle, the multidisciplinary Chest Pain Committee consisting of representation from emergency medicine physicians, cardiologists, nursing administration, hospital administration, laboratory, medical imaging and cardiovascular services researched multiple risk assessment tools. Since it is simple to use and it was designed specifically for use in the ED, the authors were most confident with the HEART Score as the tool for our institutional CP-ADP. A literature review of best practices utilizing the HEART Score identified CP-ADP protocols successfully implemented at other institutions.^5^ The Chest Pain Committee modified existing protocols to meet our institutional needs such as using three-hour troponin intervals and designing discharge forms consistent with institutional specifications.

The CP-ADP stratified patients as ‘low risk’ if the patient had a HEART score of less than 3 and had 2 negative troponin tests 3 hours apart. Once stratified as low risk, a shared decision-making process was implemented and the patient would be offered inpatient admission or discharge home with close outpatient follow-up. The shared decision-making conversation was not scripted. Rather, the launch of the CP-ADP included educating the emergency medicine providers concerning the results of previous HEART Score studies. This knowledge empowered the emergency medicine providers with the flexibility to tailor the shared decision-making conversation to the needs of individual patients.

The CP-ADP was launched in April 2016. The launch included a series of one-hour educational training sessions delivered each month for a three month period for emergency medicine physician staff, including attending and resident physicians. The training included instruction on calculating the HEART Score, methods for communicating with patients regarding their HEART score, shared decision-making processes offering the patients inpatient observation versus discharge with outpatient follow-up and instruction on utilizing the Chest Pain Discharge Form. Non-Human Subject IRB exemption was granted from the McLaren Oakland IRB for this project prior to any data collection.

As stated previously, HEART is an acronym for the components of the score: History, Electrocardiogram, Age, Risk factors, and Troponin. Troponin is a protein released into the blood when the heart muscle is damaged, such as occurs in a heart attack. Each of these components is graded as 0, 1, or 2 points. The HEART score is the sum total of these components.^2^ (Appendix 1)

The CP-ADP was used by ED physicians to stratify patients who presented to the ED with chest pain as “low risk” if their total HEART Score was 0-3 and had two negative (reference range 0-0.056 mg/dl) troponin levels three hours apart. For low-risk patients, the shared decision-making discussion was implemented.

As part of the shared decision-making process, the patients were offered the following options:

a) Inpatient hospital observation,

b) Discharge with outpatient follow up with the patient’s established primary care physician or established cardiologist within seven days, or

c) Discharge with outpatient follow up using one of two hospital-employed primary care physicians who agreed to see patients within seven days of discharge.

Regardless of discharge follow-up option chosen, patients were provided specific CP-ADP discharge instructions to return at any time if their chest pain worsened or if they changed their mind and wished to come back to receive inpatient evaluation.

All patients who chose the option for discharge under the CP-ADP between August 1, 2016 and Dec 31, 2016 (n = 50) were contacted by telephone 30 days after their ED discharge. They were asked the following three questions during post-discharge phone evaluations:

1. Had the patient experienced any MACE within the 30 days post-discharge period? (MACE events include death, myocardial infarction, or need for coronary revascularization).

2. Had the patient completed a follow up clinic appointment within seven days with a primary care physician or cardiologist?

3. Had the patient received any type of provocative cardiac testing (i.e., cardiac stress test)?

## RESULTS

During the data collection period, a total of 50 patients were discharged after being risk-stratified as “low risk” in the CP-ADP. Of these 50, 18 (36.0%) patients had non-working phone numbers or were unable to be reached by telephone after three attempts. Two (4.0%) patients opted to return to the ED for further evaluation of their chest pain and were subsequently placed in cardiac observation. One patient was in the hospital obtaining a cardiac workup at the time of contact and the other was discharged from the hospital after a negative cardiac workup three days prior to the follow up call. Neither of these patients were included in the project analyses. Therefore, a total of 30 (60% of discharged sample) patients were successfully contacted and used to evaluate the CP-ADP.

**Figure 1:**
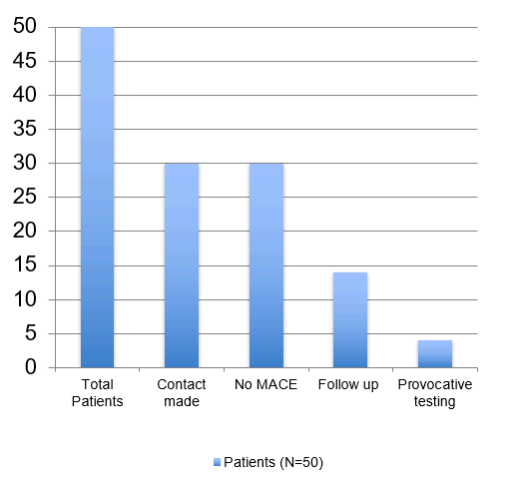
Patient Outcomes Following Discharge from the ED

Of the 30 total patients who were contacted, none (0%) experienced any MACE events. 14 (47%) of the 30 patients received outpatient follow up within seven days following discharge from the emergency room and four (13%) had received provocative cardiac testing. (Figure 1)

## DISCUSSION

Based on these project results, the HEART score is easy to learn and use and has allowed us to accurately risk-stratify patients with a chief complaint of chest pain who present to the emergency department.

Previous project results have also given us additional insight into the HEART score and chest pain in the ED. Numerous studies and articles have demonstrated the financial burden that is encountered by the workup and admission of low risk patients.^5^ In addition, physicians also inevitably feel a responsibility to ensure a safe discharge for patients who present to their ED with a chief complaint of chest pain.^4^ It is important that there are studies which can give them that reassurance to practice appropriately.

The purpose of this study was to utilize the PDSA model to evaluate and modify the CP-ADP to meet the local needs of our patients and institution. This study specifically quantified the Study, ‘S’ portion, of the PDSA cycle as part of the evaluation and modification of the CP-ADP. The McLaren Oakland CP-ADP modified its design based on published and validated ADP’s from other institutions.^5^ Based on these initial project results, low risk chest pain patients were appropriately discharged home for continued follow-up. In our study population, it was found that these low risk patients could be safely discharged with no MACE.

While the authors were encouraged by the fact that none of the patients discharged experienced MACE, these findings demonstrated a need for modification of the ADP to ensure better outpatient follow-up.

The multidisciplinary Chest Pain Committee has met multiple times discussing the data and the results of the Chest Pain ADP evaluation. After healthy discussion among the ED providers, cardiologists, primary care providers and hospital administration, a modified ‘physician directed follow-up’ is currently in the Plan stage. The physician-directed follow up consists of, with the patient’s permission, forwarding the patient’s contact information directly to the office of a hospital-employed primary care physician. The office of the primary care physician will directly contact the patient to schedule a follow-up appointment. Once the revised plan is finalized, the cycle will continue with implementation of the revised plan (DO) and evaluation of the revised plan (Study). The authors anticipate multiple future PDSA cycles of our CP-ADP to maximize the benefit of the protocol for our patients presenting to the ED with chest pain.

Secondarily, as a result of the PDSA cycle evaluation conducted in this study, patients can feel reassured that those who are stratified into low risk categories based on their HEART Score can safely follow up outpatient with their primary care physician with an acceptably low risk for MACE.

### Conflict of Interest

The authors declare no conflict of interest.

**Appendix 1:**
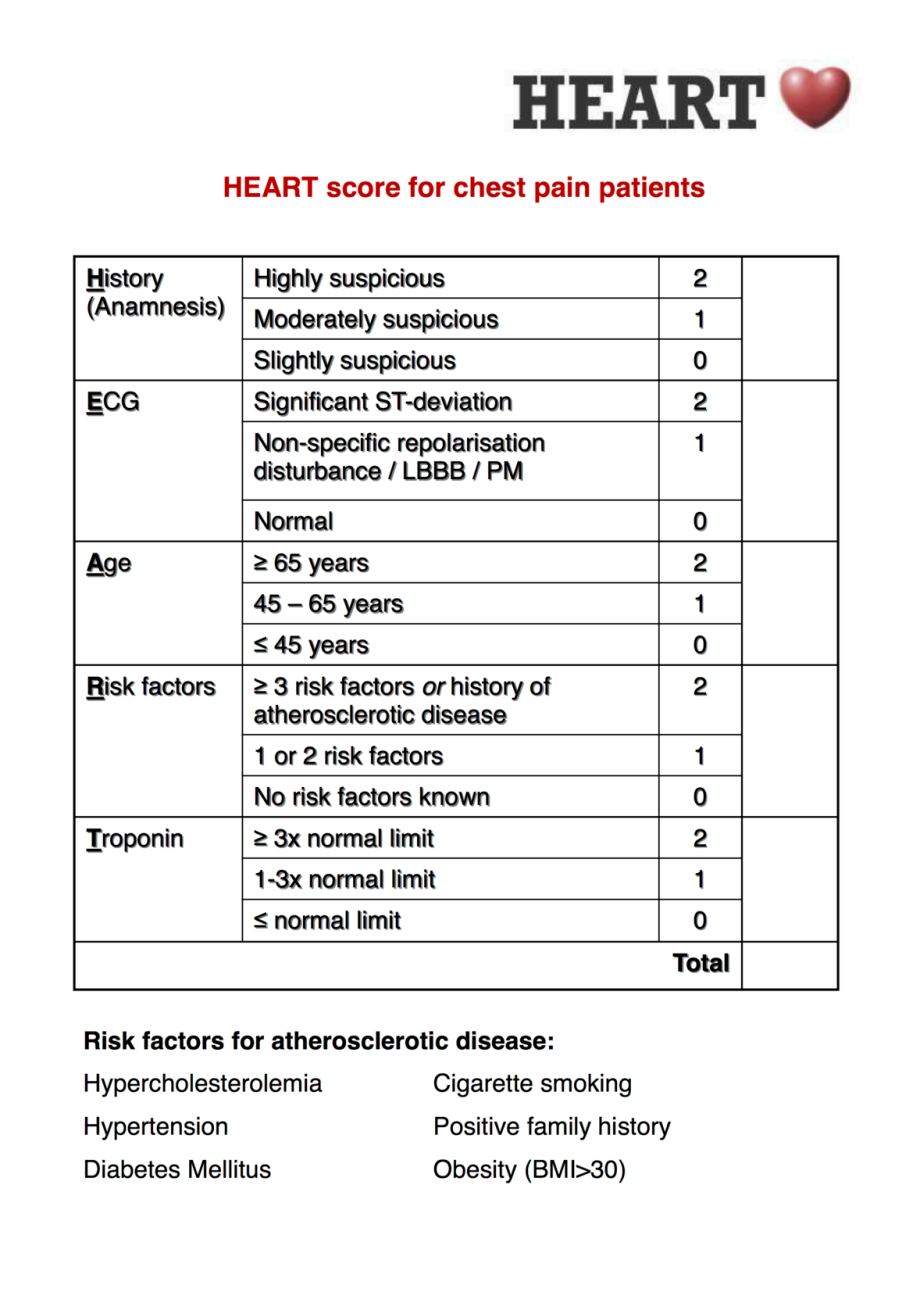
HEART Score for Chest Pain Table

**Appendix 2:**
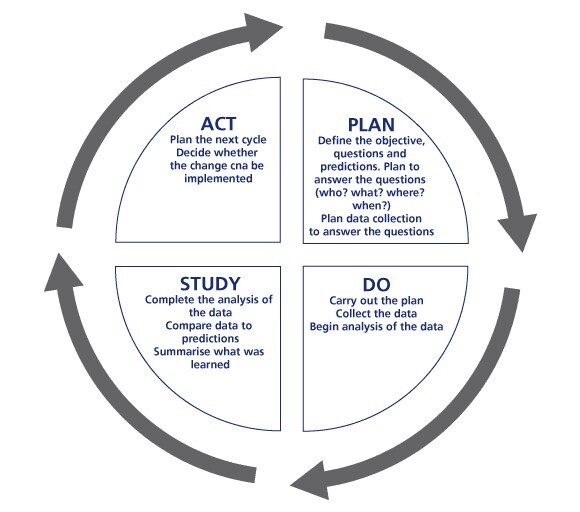
Plan-Do-Study-Act (PDSA) Model

**Appendix 3:**
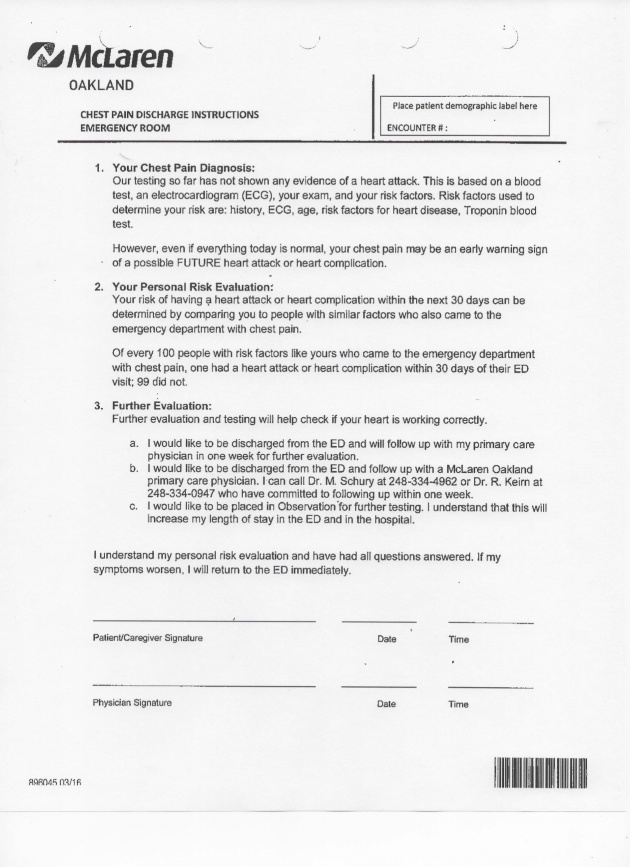
CP-ADP Discharge Form
